# Prevalence of intestinal parasitic infections and associated factors among patients attending at Sanja Primary Hospital, Northwest Ethiopia: An institutional-based cross-sectional study

**DOI:** 10.1371/journal.pone.0247075

**Published:** 2021-02-16

**Authors:** Tahir Eyayu, Teklehaimanot Kiros, Lemma Workineh, Meslo Sema, Shewaneh Damtie, Wasihun Hailemichael, Eninur Dejen, Tegenaw Tiruneh

**Affiliations:** Department of Medical Laboratory Sciences, College of Health Sciences, Debre Tabor University, Debre Tabor, Ethiopia; Universita degli Studi di Parma, ITALY

## Abstract

**Background:**

Intestinal Parasitic Infections are the most prevalent diseases in the world, predominantly in developing countries. It is estimated that more than two billion people are affected globally, mostly in tropical and sub-tropical parts of the world. Ethiopia is one of the countries in Africa with a high prevalence of intestinal parasites. However, there is a limited study conducted in the study area. Hence, this study was to assess the prevalence and associated factors of intestinal parasitosis among patients attending at Sanja Primary Hospital, Northwest Ethiopia.

**Methods:**

An institutional-based cross-sectional study was conducted at Sanja Primary Hospital from January 1 to August 20, 2019. Stool samples were collected from 1240 study participants and analyzed by direct wet mount and formal ether concentration techniques. Furthermore, sociodemographic and explanatory variables were collected using a face-to-face interview. Data were entered into Epi data version 4.4.2.1 and transferred to SPSS version 23 for analysis. Bivariate and multivariate binary logistic regression models were fitted to identify associated factors of intestinal parasitic infections. Adjusted Odds Ratio (AOR) with 95% confidence interval (CI) was considered to ascertain the significance of the association.

**Results:**

The overall prevalence of intestinal parasitic infection was 52.9% (95% CI: 50.2%-55.5%). *Entamoeba histolytica/dispar* (21.5%) was the leading cause of intestinal parasitosis followed by Hookworm species (13.3%). Furthermore, the rate of double and triple parasitic infections was observed in 6.1% and 0.5% of study participants respectively. Being Illiterate (AOR: 2.87, 95% CI: 1.06–7.47, p = 0.038), swimming habits of more than 4 times a month (AOR = 2.91, 95% CI 1.62–5.24, p< 0.001) and not washing hands before a meal (AOR: 3.92, 95% CI: 1.74–8.83, p = 0.001) were the key factors significantly associated with intestinal parasitic infection.

**Conclusions:**

The present study showed that the prevalence of intestinal parasitosis is high in the study area. Therefore, there is a need for an integrated control program, including improving personal, environmental sanitation and health education should be given to have a lasting impact on transmission.

## Background

Neglected tropical diseases, including intestinal parasitic infections (IPIs), are a highly significant cause of morbidity and mortality in endemic countries. Intestinal parasites (IPs) are present throughout the world in varying degrees of prevalence and have particular relevance as they affect the poorest and most deprived areas in tropical and subtropical regions [1, 2]. Parasitic infections caused by intestinal helminths and protozoan are among the most prevalent infections in developing countries carrying a high burden of morbidity and mortality [3]. Ascariasis, hookworm infection, amoebiasis and trichuriasis are the most common IPIs in the world [4].

The burden of diseases associated with IPI is huge. Approximately, 4.5 billion people are at risk, more than two billion people are affected worldwide, of whom 300 million suffer from associated severe morbidity [4, 5]. According to the World Health Organization report, there were 800–1000 million *Ascaris lumbricoides*, 700–900 million *Hookworm* infections, 500 million *Trichuris trichuria*, 200 million *Giardia lamblia*, and 500 million *Entamoeba histolytica/dispar* cases globally [6–9]. Based on previous estimates, the IPIs causes the annual loss of about 39 million disability-adjusted life years, which are responsible for vast health and financial problem. On the other hand, the most important drawback of IPI’s is around 90% of infected individuals remain in asymptomatic stages [10].

Epidemiological studies carried out in different countries have shown that the socio-economic level of the society and environmental factors like hot and humid tropical climate plays a role in the increment of IPIs prevalence [5, 10]. In Sub-Saharan African (SSA) countries up to 250 million people are estimated to be infected with at least one or more species of IP [11]. Intestinal parasites are highly prevalent in developing countries, including Ethiopia mainly related to poverty, poor personal hygiene, environmental sanitation, overcrowding, low level of education and lack of safe drinking water [7]. On the other hand, between one-quarter and one-third of the population in SSA countries is affected by one or more soil-transmitted helminth infections. From the total of the world’s 207 million estimated cases of schistosomiasis, 192 million (93%) occur in SSA [12]

Ethiopia is one of the poorest and least developed countries in the world with a high prevalence of IPIs. Based on the studies conducted in Ethiopia; the national prevalence of *A*. *lumbricoides*, *T*. *trichuria* and hookworm is 37%, 30%, and 16% respectively. Thus, Ethiopia has the second-highest ascariasis, the third-highest hookworm and the fourth-highest trichuriasis infections in SSA [12–14]

Many research works indicated different factors had an association with the prevalence of IPIs among suspected individuals, some of these factors related to sociodemographic characteristics and some others might be related to individuals’ practices. From sociodemographic characteristics; age, sex, residence and occupation of individuals had associated with IPIs [15–19]. Additionally, hand washing practice after toilet, absence of toilet, swimming habit, and shoe wearing habit had associated with IPIs [7, 17].

Epidemiological information regarding the prevalence and possible risk factors of IPIs on patients with gastrointestinal symptoms were not illustrated in many parts of the country including in the study area. Therefore, this study aimed to assess the prevalence of IPI and related associated factors among patients with gastrointestinal symptoms in Sanja Primary Hospital, Northwest Ethiopia.

## Methods and materials

### Study area

The study was conducted in Sanja Town, located about 65 km northwest of Gondar town and 792 km away from Addis Ababa the capital city of Ethiopia. The area has altitudinal ranges of 1900 to 2200 m with 13°20’ N 36°44’ E coordinates. The annual rainfall is 800 mm to 1800 mm, reaching its peak from July to August. It has a climatic condition of most of the area is “Kolla” and the temperature is characterized by a warm climate with a mean annual minimum temperature of 25°C and a maximum of 42°C. According to the population projection of Ethiopia in 2017, an estimated total population of Sanja Town is 12,333, of whom 5,887 were males and 6,446 were females [20]. In Sanja town, there are one hospital, one health center, and five private clinics that provide health care service. The hospital had provided diagnostic and treatment services for the inhabitants of the town and the surrounding areas.

Sanja town is traversed by one river and one stream, namely, Sanja River and Maho stream, which serve as sources of water for bathing, washing clothes, and other domestic and recreational purposes. The local people have a bad sanitary practice which includes open defecation along waterways and improper waste disposal. They can be the main sources of IPI.

### Study design, period, population and sampling technique

An institution-based cross-sectional study was conducted at Sanja primary hospital from January 01 to August 20, 2019, to determine the prevalence and associated factors of IPIs among patients attending at Sanja Primary Hospital during the study period. The study population comprised all age group individuals, who attended at Sanja Primary Hospital. The sample size was calculated using a single population proportion formula by considering a 50% prevalence of IPI, 95% confidence level, 3% margin of error, and 15% non-response rate. Accordingly, the overall estimated sample size of 1240 study participants were included in this study. The study participants were selected by a non-probability convenience sampling technique. Stool samples were received from selected study participants in order to examine the presence of IPs.

### Inclusion and exclusion criteria

All individuals with gastrointestinal symptoms and clinically suspected for IPI who presenting themselves to Sanja Primary Hospital inpatient and outpatient departments were included in the study. Intestinal parasite infections are associated with many symptoms such as diarrhea, abdominal pain or tenderness, nausea, vomiting, weight loss, indigestion/dyspepsia, bloating, constipation loss of appetite, weight loss, intestinal blood loss and others. Individuals who received anti-intestinal parasite drug/s within two weeks before data collection and those who did not provide the samples at the time of stool collection were excluded from the study.

### Data collection procedure

#### Questionnaire survey

Sociodemographic characteristics of study participants and associated factors for IPIs were collected by face-to-face interviews using a pretested structured questionnaire. To ensure the reliability of data, participants were interviewed in their mother tongue which is the Amharic language. During the interview, fingernail status and shoe wearing habits were collected through direct observations of the study participants.

#### Sample collection and laboratory analysis

Before sample collection clear orientation was given to each participant on how to collect appropriate and sufficient amounts of specimens. Then, a clean, dry, and leakproof container, which was labeled with a unique ID number was given for each participant/caregiver to collect the sample. A 5gm of stool specimen was collected from each selected study participants. All stool samples were tested in Sanja Primary Hospital laboratory as per the routine laboratory protocol, which included gross and microscopic examination within 15 minutes of its collection. In a gross examination, the color and consistency of the stool samples, presence or absence of mucus, blood, adult worms, and body segments of the parasites were physically examined. Then two medical laboratory technologists perform both wet mount and concentration techniques for further identification of IP through a light microscope. All standard procedures were strictly followed from specimen collection up to the recording and reporting of results.

*Direct microscopic examination using normal saline and iodine preparation*. About 1–2 mg of stool was emulsified in 1–2 drops of normal saline (0.9%) or Lugol’s iodine solution. A cover-slip was placed and the slide was scanned under 10× and 40× objective of a light microscope [21]. Saline wet mount smear preparation was done to detect both cyst and trophozoites of protozoa and helminth eggs or larvae. Iodine direct smear allows the examination of the characteristic features of the protozoa and the identification of the *E*. *histolytica/dispar* cyst from the commensal *E*. *coli* [21, 22].

*Formol–ether concentration technique*. Is the most frequently used technique because it concentrates a wide range of parasites with minimum damage to their morphology and it is important to increase the chance of detecting low parasite density. One gram of each sample was emulsified in 7 ml of 10% formalin solution into a centrifuge tube. Sieve the emulsified faeces and collected them in to test tube. Add 3–4 ml of diethyl ether and mixed for 15 seconds. The formol-ether emulsion was then centrifuged at 1500 rpm for 1 minute. Then the centrifuge tube inverted for the supernatant to be decanted off, allowing a few drops of the deposit to remain. This was well mixed and a drop was placed on a clean glass slide. The slide was covered using a cover-slip and examined under 10× and 40× objective of a light microscope [21, 22].

### Data quality control

Initially, the study tool (questionnaire) was prepared in the English language (S1 File). Then, it was converted into the Amharic language (S2 File). Lastly, it was retranslated back to English to retain its accuracy and consistency. The evaluation was conducted to consider the consistency and accuracy of the two types of study tools. The pre-test was conducted on 5% of samples in Koladiba’s primary hospital other than the actual study area. To maintain the quality of data, appropriate training was given for data collectors. For each stool sample, two slides were prepared and examined by different laboratory technologists All standard operating procedures were strictly followed during the stool sample examination to ensure the quality of the test result. Moreover, all reagents were stored and prepared according to the manufacturer’s instructions.

### Data management and analysis

Data were coded and entered into EpiData Manager version 4.4.2.1 statistical software and then exported to SPSS version 23 for analysis. Descriptive statistics were used to summarize the sociodemographic characteristics of the study participants. Binary logistic regression analyses were used to assess the association between explanatory variables and the outcome variable. Firstly, bivariate binary logistic regression analysis was done, and then to control the possible confounding, variables with a *p*-value < 0.2 were adjusted by multivariate logistic by stepwise variable selection. The strength of the association between predictor and outcome variables were assessed by using the adjusted odds ratio (AOR) and 95% confidence interval (CI). In all cases, *p*-value ≤ 0.05 was considered as a statistically significant association.

#### Ethical considerations

The research was conducted after obtaining an ethical clearance letter from the School of Biomedical and Laboratory Sciences Research and Ethical Review Committee (reference no. SBMLS/2123/11). A permission letter was obtained from woreda health office and clinical director of Sanja primary hospital. Informed written consent was obtained from each study participant and parents/legal guardians of children. Information collected from the study participants was kept confidential. Any individuals who were positive for different IPIs were linked to the responsible body in Sanja Primary Hospital for treatment. This study was conducted in accordance with the declaration of Helsinki.

## Results

### Socio-demographic characteristics of study participants

A total of 1240 participants with gastrointestinal symptoms who were attending at Sanja Primary Hospital within the study period were included in the present study. Of the total participants, 50.3% were males while the remaining 49.7% were females. The age of the study participants was ranged from 1 to 69 years with a mean age of 28.51 (SD±14.73) years. More than half of the study participants were between the age of 15–44 years. A majority of them were orthodox in religion, 91.8%, living in rural areas 53.4%, and farmers 25.6%. Regarding marital status, 52.4% were married. Based on occupation the least percentage accounted for the governmental employees (Table 1).

**Table 1:** Socio-demographic characteristics of study participants with gastrointestinal symptoms attending at Sanja Primary Hospital, Northwest Ethiopia, 2019.

Characteristics (n = 1240)	Categories	Frequency (n)	Percent (%)
**Sex**	Male	624	50.3
	Female	616	49.7
**Age in years**	≤ 14	204	16.5
	15–29	514	41.5
	30–44	332	26.8
	≥ 45	190	15.3
**Residence**	Urban	578	46.6
	Rural	662	53.4
**Religion**	Orthodox	1138	91.8
	Muslim	102	8.2
**Occupation**	Student	280	22.6
	Unemployed	85	6.9
	Daily laborer	124	10.0
	Housewife	299	24.1
	Farmer	317	25.6
	Merchant	103	8.3
	Government employee	32	2.6
**Educational status**	Illiterate	315	25.4
	Only read and write	230	18.5
	Primary school	393	31.7
	Secondary school	267	21.5
	Diploma and above	35	2.8
**Marital status**	Single	512	41.3
	Married	650	52.4
	Divorced	51	4.1
	Widowed	27	2.2

### Prevalence of intestinal parasitic infections

The overall prevalence of IPI in Sanja Primary hospital was 52.9% (95% CI: 50.2%-55.5%) which is observed in 656 suspected individuals. Different types of parasites, including protozoans, nematodes, trematodes and cestodes were detected from the stool samples of study participants. Out of the 1240 study participants examined, 346 (27.9%) were found to be infected with one or more intestinal protozoa, 282 (22.7%) were infected with helminths and 28 (2.3%) had mixed infection with both protozoa and helminths.

In this study, eight different species of IP were identified, two protozoans, and six helminth species. Of these detected parasites, *E*. *histolytica/dispar*, 21.6% (268/1240) was the most common parasite followed by *Hookworm species* 13.3% (165/1240), *Schistosoma mansoni* 9.4% (116/1240), *G*. *lamblia* 9.2% (114/1240), *A*. *lumbricoides* 2.1% (26/1240), *Entrobious vermicularis* 0.5% (6/1240), *Hemonolopus nana* 0.3% (4/1240), and *Taenia species* 0.2% (3/1240) singly or mixed with other parasites. Based on the microscopic examination of stool specimens using different methods the prevalence of detected IP is embedded in Table 2.

**Table 2:** Distribution of intestinal parasite species among study participants (n = 1240) at Sanja Primary Hospital, Northwest Ethiopia, 2019.

Type of intestinal parasite detected	Number of infected	Prevalence (95%CI)
**Single infection (n = 613)**		
*E*. *histolytica/dispar*	245	19.8 (17.5 to 22.0)
*Hookworm species*	139	11.2 (9.5 to 13.1)
*S*. *mansoni*	96	7.8 (6.3 to 9.2)
*G*. *lamblia*	94	7.6 (6.2 to 9.1)
*A*. *lumbricoides*	26	2.1 (1.3 to 3.0)
*E*. *vermicularis*	6	0.5 (0.2 to 0.9)
*H*. *nana*	4	0.3 (0.1 to 0.6)
*Taenia species*	3	0.2 (0.0 to 0.6)
**Double infection (n = 40)**		
*E*. *histolytica/dispar* and *Hookworm species*	8	0.6 (0.2 to 1.1)
*Hookworm species* and *S*. *mansoni*	8	0.6 (0.2 to 1.1)
*Hookworm species* and *G*. *lamblia*	8	0.6 (0.2 to 1.1)
*E*. *histolytica/dispar* and *G*. *lamblia*	7	0.6 (0.2 to 1.0)
*S*. *mansoni* and *E*. *histolytica/dispar*	5	0.4 (0.1 to 0.8)
*S*. *mansoni* and *G*. *lamblia*	4	0.3 (0.1 to 0.6)
**Triple infection (n = 3)**		
*E*. *histolytica/dispar*, *Hookworm species* and *S*. *mansoni*	2	0.2 (0.0 to 0.4)
*E*. *histolytica/dispar*, *S*. *mansoni* and *G*. *lamblia*	1	0.1 (0.0 to 0.2)
**Total**	**656**	**52.9 (50.2 to 55.5)**

CI: Confidence interval.

Regarding the number of parasite species detected in each sample, the majorities of the positive cases were single infection 93.4%. Furthermore, double and triple parasitic infections were observed in 6.1% and 0.5% of IP infected participants respectively. Of the triple infected persons, two were coinfected with *E*. *histolytica/dispar*, *Hookworm species* and *S*. *mansoni*. And the other one with *E*. *histolytica/dispar*, *S*. *mansoni* and *G*. *lamblia* infection.

### Analysis of associated factors for intestinal parasitic infections

In this study bivariable and multivariable binary logistic regression was used to determine associated factors of IPTs. In this study, several factors were considered to assess factors that contribute to IPIs (see Table 3). From sociodemographic factors; age group of ≤ 14 years and residence was significantly associated with the presence of IP in bivariable binary logistic regression. But sex, religion, occupation, educational level and marital status of individuals were not statistically significant in bivariate binary logistic regression analysis.

**Table 3:** Bivariate and multivariate binary logistic regression analysis of factors associated with IPIs at Sanja Primary Hospital, Northwest Ethiopia.

Variables		IPI		COR (95% CI)	AOR (95% CI)
		No Positive	No Negative		
**Sex**	Male	338 (54.2%)	286 (45.8%)	1.11 (0.89–1.38)	
	Female	318 (51.6%)	298 (48.4%)	1.0	
**Age in years**	≤ 14	119 (58.3%)	85 (41.7%)	1.49 (1.00–2.22)	1.62 (0.91–2.88)
	15–29	280 (54.5%)	234 (45.5%)	1.28 (0.91–1.78)	**1.72** (**1.13–2.63**)*
	30–44	165 (49.7%)	167 (50.3%)	1.05 (0.74–1.50)	1.22 (0.82–1.83)
	≥ 45	92 (48.4%)	98 (51.6%)	1.0	1.0
**Residence**	Urban	276 (47.8%)	302 (52.2%)	1.0	1.0
	Rural	380 (57.4%)	282 (42.6%)	1.47 (1.18–1.85)	0.79 (0.57–1.10)
**Occupation**	Student	160 (57.1%)	120 (42.9%)	1.95 (0.93–4.10)	1.02 (0.38–2.70)
	Unemployed	44 (51.8%)	41 (48.2%)	1.57 (0.69–3.58)	0.47 (0.15–1.47)
	Daily labor	62 (50.0%)	62 (50.0%)	1.46 (0.66–3.22)	0.71 (0.26–1.95)
	Housewife	153 (51.2%)	146 (48.8%)	1.53 (0.73–3.21)	0.75 (0.29–1.96)
	Farmer	173 (54.6%)	144 (45.4%)	1.76 (0.84–3.68)	0.69 (0.26–1.82)
	Merchant	51 (49.5%)	52 (50.5%)	1.43 (0.64–3.20)	1.02 (0.38–2.75)
	Government employee	13 (40.6%)	19 (59.4%)	1.0	1.0
**Religion**	Orthodox	601 (52.8%)	537 (47.2%)	0.96(0.64–1.44)	
	Muslim	55 (53.9%)	47 (46.1%)	1.0	
**Educational status**	Illiterate	179 (56.8%)	136 (43.2%)	1.97 (0.97–4.02)	**2.87** (**1.06–7.47**)*
	Only read and write	131 (57.0%)	99 (43.0%)	1.99 (0.96–4.10)	2.30 (0.89–5.96)
	Primary	210 (53.4%)	183 (46.6%)	1.72 (0.85–3.48)	1.52 (0.60–3.87)
	Secondary	122 (45.7%)	145 (54.3%)	1.26 (0.62–2.59)	1.12 (0.45–2.80)
	Diploma and above	14 (40.0%)	21 (60.0%)	1.0	1.0
**Marital status**	Single	290 (56.6%)	222 (43.4%)	0.90 (0.41–1.97)	
	Married	322 (49.5%)	328 (50.5%)	0.68 (0.31–1.48)	
	Divorced	28 (54.9%)	23 (45.1%)	0.84 (0.33–2.15)	
	Widowed	16 (59.3%)	11 (40.7%)	1.0	
**Contact to river when crossing**	Yes	354 (54.2%)	299 (45.8%)	1.12 (0.89–1.40)	
	No	302 (54.4%)	285 (45.6%)	1.0	
**Wash cloth on river**	Yes	373 (57.2%)	279 (52.8%)	1.44 (1.15–1.80)	1.25 (0.98–1.60)
	No	283 (48.1%)	305 (51.9%)	1.0	1.0
**Irrigation activities**	Yes	55 (56.7%)	42 (43.3%)	1.18 (0.78–1.79)	
	No	601 (52.6%)	542 (47.4%)	1.0	
**Swim in river**	Yes	250 (57.7%)	183 (42.3%)	1.35 (1.07–1.71)	1.35 (1.05–1.74)*
	No	406 (50.3%)	401 (49.7%)	1.0	1.0
**Swimming per month**	None	406 (50.3%)	401 (49.7%)	1.0	1.0
	1–2 times/month	109 (51.7%)	102 (48.3%)	1.06 (0.78–1.43)	1.01 (0.73–1.39)
	3–4 times/month	90 (58.8%)	63 (41.2%)	1.41 (0.99–2.00)	1.34 (0.92–1.95)
	>4 times/month	51 (73.9%)	18 (26.1%)	2.80 (1.61–4.87)	**2.91** (**1.62–5.24**)**
**Hand wash after defecation**	Always	201 (45.0%)	246 (55.0%)	1.0	1.0
	Sometimes	233 (56.4%)	180 (43.6%)	1.58 (1.21–2.07)	**1.54** (**1.16–2.05**)**
	Nor at all	222 (58.4%)	158 (41.6%)	1.72 (1.30–2.27)	**1.58** (**1.17–2.12**)**
**Hand wash before a meal**	Always	213 (43.6%)	275 (56.4%)	1.0	1.0
	Sometimes	331 (57.5%)	245 (42.5%)	1.74 (1.37–2.23)	**2.33** (**1.42–3.83**)**
	Nor at all	112 (63.6%)	64 (36.4%)	2.26 (1.58–3.22)	**3.92** (**1.74–8.83**)**
**Shoe wearing habit**	Always	230 (46.1%)	269 (53.9%)	1.0	1.0
	Sometimes	324 (56.3%)	251 (43.7%)	1.51 (1.19–1.92)	0.61 (0.37–1.00)
	Nor at all	102 (61.4%)	64 (38.6%)	1.86 (1.30–2.67)	0.44 (0.19–1.01)
**Latrine utilization**	Always	195 (44.3%)	245 (55.7%)	1.0	1.0
	Sometimes	203 (55.6%)	162 (44.4%)	1.57 (1.19–2.08)	**1.49** (**1.10–2.01**)**
	Nor at all	258 (59.3%)	177 (40.7%)	1.83 (1.40–2.40)	**1.47** (**1.07–2.03**)*
**Finger nail status**	Trimmed	316 (50.7%)	307 (49.3%)	1.0	1.0
	Not trimmed	340 (55.1%)	277 (44.9%)	1.19 (0.95–1.49)	1.06 (0.83–1.36)
**Eating raw vegetable**	Always	148 (56.9%)	112 (43.1%)	1.30 (0.94–1.78)	1.35 (0.97–1.89)
	Sometimes	311 (52.7%)	279 (47.3%)	1.09 (0.85–1.41)	1.11 (0.85–1.45)
	Nor at all	197 (50.5%)	193 (49.5%)	1.0	1.0
**Eating raw meat**	Yes	241 (53.7%)	208 (46.3%)	1.05 (0.83–1.32)	
	No	415 (52.5%)	376 (47.5%)	1.0	
**Source of water**	Pipe	209 (47.8%)	228 (52.2%)	1.0	1.0
	Well	255 (52.0%)	235 (48.0%)	1.18 (0.91–1.53)	1.04 (0.78–1.37)
	Stream	163 (60.1%)	108 (39.9%)	1.65 (1.21–2.24)	1.34 (0.96–1.87)
	River	29 (69.0%)	13 (31.0%)	2.43 (1.23–4.81)	1.77 (0.86–3.64)

**Note**:

*p-value <0.05,

**p-value <0.01; **AOR**: Adjusted Odds Ratio; **COR**: Crude Odds Ratio; **CI**: Confidence interval.

In bivariate binary logistic regression analysis, the potential associated factors such as washing clothes in the river, swimming habit, hand washing after defecation and before eating a meal, shoe-wearing habit, latrine utilization, and water source were found to be statistically associated with IPI prevalence (p-value <0.05). Multivariable logistic analysis was conducted after adjusting variables which were p-value <0.2 in the bivariate binary logistic analysis. The multivariable logistic regression model estimated that individuals within the age group of 15–29 years were 1.72 times (AOR: 1.72, 95% CI: 1.13–2.63, p = 0.012) more likely to get infected with IP than those within the age group of more than 44 years. Besides, the participant’s educational level was statistically associated with IPI. Being illiterate individuals were 2.87 times (AOR: 2.87, 95% CI: 1.06–7.47, p = 0.038) more likely to be infected with IP than those who had a diploma and above. The odds of being infected by IP among participants who swam was 1.35 more likely as compared to those who did not (AOR: 1.35 2.8, 95% CI: 1.05–1.74, p = 0.020). And also, participants with a swimming habit of more than 4 times a month were 2.91 times (AOR = 2.91, 95% CI 1.62–5.24, p < 0.001) more likely as compared to non-swimmers.

Moreover, IPI was significantly associated with lack of hand washing habit after defecation, with higher infection among infrequently washed their hands after defecation (AOR: 1.54, 95% CI: 1.16–2.05, p = 0.003) and those who do not wash their hands after defecation (AOR: 1.58, 95% CI: 1.17–2.12, p = 0.003). Those individuals who did not wash their hands before eating a meal were 3.92 more likely to be infected than those who always washed their hands before eating a meal (AOR: 3.92, 95% CI: 1.74–8.83, p = 0.001), and the study participants who washed sometimes were 2.33 more likely to be infected than those who washed always (AOR: 2.33, 95% CI: 1.42–3.83, p = 0.001) (Table 3).

## Discussion

The study indicated that more than half of study participants in Sanja primary hospital had IPIs and mixed infection with poly parasites was also common. The prevalence of IPI was 52.9% (95% CI: 50.2%-55.5%), with the leading prevalence of *E*. *histolytica/dispar* (21.5%) was the most common parasite followed by *Hookworm species* (13.3%), *S*. *mansoni* (9.4%), and *G*. *lamblia* (9.2%) in this study. The prevalence of IPIs observed in this study is comparable with the previously reported 52.47% in Burkina Faso [23] that supports our findings. The result of this study confirmed that IPIs are prevalent among patients with gastrointestinal symptoms attending at Sanja Primary Hospital and in its catchment area.

However, the current study showed a lower prevalence of IPI than the previous findings from Shahura Health Center (56.9%) [15], Tseda Health Center (62.3%) [7] Northwest Ethiopia and the University hospital of Bobo-Dioulasso, Burkina Faso (65.3%) [24]. The possible explanation for the low prevalence of IPI in this study might be due to differences in the study period, geographic locations and the effect of enhanced outreach strategy in our country; one of the objectives of the initiative was to deworm all under five years children every six months to decrease IPI by providing anti-helminthics drugs.

On the other hand, the prevalence seen in this study was higher than those of local studies conducted in Ethiopia, such as the prevalence of IPI at Adwa health center (33.5%) [16], Red Cross Clinic and Chelaleki Health Center (37.8%) [17], and Workmeda Health Center (27.7%) [25]. The possible reason for this discrepancy might be due to sample size variations. In this study, 1240 study participants were included, which is 2 up to 3 times higher than the above listed studies. It is well known that a warmer temperature generally increases the rates of parasite development, facilitate higher rates of infectivity and heavier burdens of disease [26]. Our study was conducted in the temperate region with an average temperature range from 25 °C to 42 °C, which could be the possible reason for the high prevalence of IPIs in this study.

In addition to the above, the current IPI prevalence was higher than the studies conducted in different countries, such as reports from Northeast Nigeria (17.5%) [27], North-Western Nigeria (11.8%) [28], Northwestern Saudi Arabia (45.38%) [18], Iran (31.2%) [19], Nepal (30.1%) [29] and India (6.63%) [30]. The possible reason for this discrepancy in the prevalence of IPI across studies might be due to the differences in practices of personal hygiene, environmental sanitation, health promotion practices, climatic conditions, geographic locations and socio-economic status of the populations.

In this study, the predominant parasitic infection was *E*. *histolytica/dispar*, followed by *Hookworm species* when compared to other detected IP. This is consistent with the finding from Shahura Health Center, Northwest Ethiopia [15], Northeast Nigeria [27] and Nepal [29] those showed the most prevalent species of IP was *E*. *histolytica/dispar* followed by *Hookworm species*. The prevalence of *E*. *histolytica/dispar* in the present study was 21.6%. This result was in line with a report from the University hospital of Bobo-Dioulasso, Burkina Faso (23.4%) [24]. However, it is higher as compared to a study conducted in Northwest Ethiopia (4.6%) [7], North Ethiopia (13.6%) [16], East Wollega Zone, Ethiopia (16.3%) [17] and Northeast Nigeria (7.0%) [27]. Contamination of potable water, poor handling of food and poor hand washing habit after defecation and before a meal may be the possible reason for the high prevalence of *E*. *histolytica/dispar* infection.

In the present study, the rate of double infections was 6.1% (40/656). This is relatively similar to different findings conducted in Ethiopia (5.3%) [15, 16]. But the rate of double infection is lower as compared to previous findings from Nepal (9.6%) [29]. Variations in the level of environmental contamination and socioeconomic factors might be responsible for the difference in the prevalence of multiple parasitic infections at a time. Stool samples were processed by modified acid-fast staining method, whereas diarrheal samples were also subjected to Sheather’s sucrose flotation techniques in the previous study may be important for detecting higher rates of double infection. Additionally, the rate of triple infections in our study was 0.5% (3/656). The finding of this study was corroborated by the reports from Adwa health center, Ethiopia (0.8%) [16].

In this study, several possible risk factors associated with IPIs were investigated. Intestinal parasitic infections were significantly associated with age group, educational status, swimming habit, number of swimming per month, hand wash habit before a meal and after defecation, and latrine utilization of study participants. From which participants with a swimming habit of more than 4 times a month and not washing hands before the meal were the key factors significantly associated with IPIs in the present study.

According to the present study age group of study participants belonging to 15–29 years of old was a significant factor for IPIs. This was in agreement with a study conducted in East Wollega Zone, Ethiopia [17]. The possible reasons might be due to playing with soil and water bodies, frequent contact among each other, over-crowding in the classroom, and their involvement in farming activities at this age level facilitates the spread of the parasites.

The current study showed that the participant’s educational level was statistically associated with IPI. Illiterate individuals were 2.87 times more likely to be infected with IPIs than those who had a diploma and above (AOR: 2.87, 95% CI: 1.06–7.47, p = 0.038). This was in line with other studies conducted in Bahir Dar, Ethiopia [31] and North-Western Nigeria (11.8%) [28]. This finding might be due to a lack of awareness in the prevention and control mechanisms of IPI among illiterate individuals. Most of the time a higher level of education is associated with higher levels of hygiene and sanitation practice which might reduce the prevalence of IPI.

The practice of swimming was one of the significantly associated variables with parasitic infection (Table 3). Based on the finding of this study, Suspected individuals who had a habit of swam were 1.35 more likely to be infected with IP as compared to those who did not (AOR: 1.35 2.8, 95% CI: 1.05–1.74, p = 0.020). Similarly, a study conducted in Northwest Ethiopia showed swimming habits had shown a strong association with parasitic infection [7]. And also, study participants with a swimming habit of more than 4 times a month were 2.91 times more likely to be infected with IPIs when compared with non-swimmers (AOR = 2.91, 95% CI 1.62–5.24, p< 0.001). The possible explanation may be due to the fact that frequent contact with unprotected water bodies could result in infection through swallowing infective stage contaminated unclean water orally and skin penetrating infections.

In general, IPIs were significantly associated with poor hand washing practice. Our result showed that study participants who had infrequently washed and do not wash their hands after defecation were more than 1.5 times more likely to be infected by IP as compared to individuals who regularly wash their hands after defecation. This finding is consistent with different studies conducted in Ethiopia [7, 17]. In this study, 39.4% of the study participants practice good hand washing before eating a meal. The likelihood of acquiring infections among study participants who do not practice hand washing before eating a meal was 3.92 (AOR: 3.92, 95% CI: 1.74–8.83, p = 0.001) times and the patients who sometimes washed their hands before eating a meal was 2.33 (AOR: 2.33, 95% CI: 1.42–3.83, p = 0.001) times higher than individuals who had good hand washing practice. Unlike the current study, research work in Tseda and Shahura Health Centers, Northwest Ethiopia [7, 15] did not show a significant association between hand washing practice before eating a meal with a prevalence of IPI.

Lastly, the study stated that participants who were unable to utilize latrines properly were susceptible to be infected by IP. This finding was similar with the previous study conducted in Northwest Ethiopia [4, 7]. These results indicate that improper latrines utilization and open defecation practice contaminates the environment and water sources that play a bold role in the transmission of IP.

### Limitations of the study

Due to resource constraints, we did not perform molecular techniques like PCR to identify and discriminate the true pathogenic *E*. *histolytica* from *E*. *dispar*. Thus, overdiagnosis of amoebiasis might occur. In addition, the study didn’t include the intensity of infections for soil-transmitted helminth and *S*. *mansoni* infections.

## Conclusion

In general, the present study showed a high prevalence of IPIs in the study area indicated that parasitic infections are considerable public health problems. The present study has also revealed that *E*. *histolytica/dispar* a common parasite species that causes infection in the study area. Intestinal parasite infections were strongly associated with educational status, swimming habits, hand wash habits before a meal and after defecation.

Therefore, the authors recommend that need of integrated control program including improve personal and environmental sanitation, providing of clean potable water, periodic deworming, construction of public toilets, instilling health education, creating awareness regarding the impact of swimming in contaminated water and the importance of washing hands can be good approaches to control these infections in the area. Local government authorities must implement preventive strategies as well as regular mass deworming programs. We believe that the epidemiological data generated in this study may help local and regional health authorities and decision makers to optimize and implement resources to improve the quality of life and combat intestinal parasite infections.

## Supporting information

S1 File English version questionnaire. (DOCX)

S2 File Amharic version questionnaire. (DOCX)

## Abbreviations

°C: Degree Celsius
AOR: Adjusted Odds Ratio
CI: Confidence Interval
COR: Crude Odds Ratio
IP: Intestinal Parasites
IPI: Intestinal Parasitic Infection
SSA: Sub-Saharan African
