# Supplementary material for: Prevalence of intestinal parasitic infections and associated factors among patients attending at Sanja Primary Hospital, Northwest Ethiopia: An institutional-based cross-sectional study
Source: PLoS One. 2021 Feb 16;16(2):e0247075. doi: 10.1371/journal.pone.0247075 (PMC7886201; doi:10.1371/journal.pone.0247075)
Supplement: S1 File — (DOCX) [file pone.0247075.s001.docx]

Date___________________

**English version questionnaire**

Dear Study participants my name is _______________. I am a lecturer and researcher from Debre Tabor University. I am doing the study to figure out the Prevalence of intestinal parasitic infections and associated factors among patients attending at Sanja primary hospital, Northwest Ethiopia. I want to assure you that all of your answers will be kept strictly and only used for research purpose. I humbly ask you to do his part to pass on the right information. If you are willing to participate, we will ask you.

**A. Yes, I agree B. I do not agree**

**Participant identification:** Code No ________________ Serial No _______________

**Part I – Socio-demographic characteristics of study subjects**

| **S. No** | **Questions or characteristics** | **Answers and codes** | **Skip** |
| --- | --- | --- | --- |
| 101 | Sex | 1. Male 2. Female |  |
| 102 | How old are you? | Age -------------------- |  |
| 103 | Residence | 1. Urban 2. Rural |  |
| 104 | What is your occupation? | 1. Student 2. Unemployed 3. Daily labor 4. House wife 5. Farmer 6. Merchant 7. Government employee 8. Others (specify)____________ |  |
| 105 | What is your religion? | 1. Orthodox 2. Protestant 3. Muslim |  |
|  |  | 1. Other (specify)_____________ |  |
| 106 | What is your educational status? | 1. Illiterate 2. Only read and write 3. Primary (grade 1-8) 4. Secondary (grade 9-12) 5. Diploma and above |  |
| 107 | What is your current Marital status? | 1. Single  2. Married  3. Divorced  4. Widowed |  |

**Part II. Associated factors for intestinal parasites**

| **S. No** | **Questions or characteristics** | **Answers and codes** | **Skip** |
| --- | --- | --- | --- |
| 201 | Do you have contact to river while you are crossing it? | 1. Yes 2. No |  |
| 202 | Do you wash your cloth on the river? | 1. Yes 2. No |  |
| 203 | Do you participate in irrigation? | 1. Yes 2. No |  |
| 204 | Do you swim in the River? | 1. Yes 2. No | If no go to 206 |
| 205 | How many times per a month do you swim in the river? | 1. 1-2 times/month 2. 3-4 times/month 3. >4 times/month |  |
| 206 | Hand washing habit after defecation? | 1. Always 2. Sometimes 3. Nor at all |  |
| 207 | Hand washing habit before meal? | 1. Always 2. Sometimes 3. Nor at all |  |
| 208 | Shoe wearing habit | 1. Always 2. Sometimes 3. Nor at all |  |
| 209 | Habit of latrine utilization | 1. Always 2. Sometimes 3. Nor at all |  |
| 210 | Finger nail status | 1. Trimmed 2. Not trimmed |  |
| 211 | Habit of eating raw vegetable | 1. Always 2. Sometimes 3. Nor at all |  |
| 212 | Do you have the habit of eating raw meat? | 1. Yes 2. No |  |
| 213 | Source of drinking water | 1. Pipe 2. Well 3. Stream 4. River 5. Other (specify) ________ |  |
