# Supplementary material for: Prevalence of intestinal parasitic infections and associated factors among patients attending at Sanja Primary Hospital, Northwest Ethiopia: An institutional-based cross-sectional study
Source: PLoS One. 2021 Feb 16;16(2):e0247075. doi: 10.1371/journal.pone.0247075 (PMC7886201; doi:10.1371/journal.pone.0247075)
Supplement: S2 File — (DOCX) [file pone.0247075.s002.docx]

ቀን___________________

**አማርኛ ቃለ-መጠይቅ**

ዉድ የጥናቱ ተሳታፊዎች ስሜ **_________________** ይባላል፣ የደብረታቦር ዩኒቨርሲቲ መምህርና ተመራማሪ ነኝ፡፡ በአሁኑ ጊዜ በሰሜን ምዕራብ ኢትዮጵያ በሳንጃ የመጀመሪያ ደረጃ ሆስፒታል በሚከታተሉት ህመምተኞች ላይ የአንጀት ጥገኛ ተህዋሲያን ስርጭት እና ተያያዥ ምክንያቶች ላይ ጥናት እየሰራሁ እገኛለሁ። ስለሆነም እርስዎ ትክክለኛ መረጃ በመስጠት የበኩለዎትን እንዲወጡ ስል በትህትና እየጠየኩ መረጃዋቹ ከጥናቱ አላማ ውጭ እንደማይውሉ እና ሚስጥራቸውም በጥብቅ እንደሚጠበቅ ቃል እየገባው ፈቃደኛ ከሆኑ ወደ ጥያቄው እንገባለን፡፡

ሀ. አዎ ፍቃደኛ ነኝ ለ. ፈቃደኛ አይደለሁም

የተሳታፊ መለያ: የሚስጥር ቁጥር________________ መለያ ቁጥር:_________________

**ክፍል I - የማኅበራዊና ግላዊ መረጃን የተመለከተ መጠይቅ**

| **መ.ቁ** | **ጥያቄዎችና መለያዎች** | **መልስና ኮድ** | **ይለፍ** |
| --- | --- | --- | --- |
| 101 | ጾታ | 1. ወንድ 2. ሴት |  |
| 102 | እድሜዎት ስንት ነው? | ዕድሜ_____________________ |  |
| 103 | የመኖሪያ አድራሻ | 1. ከተማ 2. ገጠር |  |
| 104 | የእርስዎ የስራ አይነት ምንድን ነው? | 1. ተማሪ 2. ስራ የሌለው 3. የቀን ሰራተኛ 4. የቤት እመቤት 5. ገበሬ 6. ነጋዴ 7. የመንግስት ሰራተኛ |  |
|  |  | 1. ሌላ ካለ ይጠቀስ____________ |  |
| 106 | ሀይማኖትዎ ምንድን ነው? | 1. ኦርቶዶክስ 2. ፕሮቴስታንት 3. ሙስሊም |  |
|  |  | 1. ሌላ ካለ ይጠቀስ____________ |  |
| 107 | የእርስዎን የትምህርት ደረጃ ምንድን ነው? | 1. ያልተማሩ 2. ማንበብና መጻፍ የሚችል 3. የመጀመሪያ ደረጃ (1-8) |  |
|  |  | 1. ሁለተኛ ደረጃ (9-12) 2. ዲፕሎማ እና ከዛ በላይ |  |
| 108 | የትዳር ሁኔታ | 1. ያላገባ/ች 2. ያገባ/ች 3. የፈታ/ች 4. ባል/ሚስት የሞተባቸው |  |

**ክፍል II -** **የጥናቱ ተሳታፊዎች ለአንጀት ጥገኛ ተህዋሲያን** **ያላቸው ተጋላጭነት**

| **መ.ቁ** | **ጥያቄዎችና መለያዎች** | **መልስና ኮድ** | **ይለፍ** |
| --- | --- | --- | --- |
| 201 | በወንዝ ሲያልፉ ከወንዙ ጋር ይነካካሉ? | 1. አዎ 2. አልነካካም |  |
| 202 | ልብስዎን ወንዝ ላይ ያጥባሉ? | 1. አዎ 2. አላጥብም |  |
| 203 | የመስኖ ስራ ላይ ይሳተፋሉ? | 1. አዎ 2. አልሳተፍም |  |
| 204 | ወንዝ ላይ ዋኝተው ያውቃሉ? | 1. አዉቃለዉ 2. አላዉቅም 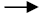 | 206 |
| 205 | በወር ለምን ያህል ጊዜ ይዋኛሉ? | 1. 1-2 ጊዜ 2. 3-4 ጊዜ 3. >4 ጊዜ |  |
| 206 | ሽንት ቤት ከተጠቀሙ በኋላ እጅን የመታጠብ ልማድ? | 1. ሁልግዜ 2. አንዳንዴ 3. አልታጠብም |  |
| 207 | ምግብ ከመብላትዎ በፊት እጅን የመታጠብ ልማድ? | 1. ሁልግዜ 2. አንዳንዴ 3. አልታጠብም |  |
| 208 | ጫማ የመልበስ ልምድ | 1. ሁልግዜ 2. አንዳንዴ 3. አላደርግም |  |
| 209 | የመፀዳጃ ቤት አጠቃቀም | 1. ሁልግዜ 2. አንዳንዴ 3. አልጠቀምም |  |
| 210 | የጣት ጥፍር ሁኔታ | 1. ተከርክሟል 2. አልተከረከመም |  |
| 211 | ያልበሰለ አትክልት የመመገብ ልማድ? | 1. ሁልግዜ 2. አንዳንዴ 3. አልመገብም |  |
| 212 | ጥሬ ስጋ የመመገብ ልምድ አለዎት? | 1. አዎ 2. የለኝም |  |
| 213 | የመጠጥ ውሃ መገኛ | 1. የቧንቧ ውሃ 2. የምንጭ ውሃ 3. ትንሽ ወንዝ 4. ወንዝ 5. ሌላ ካለ ይጠቀስ_______ |  |
